# Our Duty as Physicians

**Published:** 1897-01

**Authors:** Robert E. Moss

**Affiliations:** San Antonio, Texas


For the Texas Medical Journal.

### OUR DUTY AS PHYSICIANS.

BY ROBERT E. MOSS, M. D., SAN ANTONIO, TEXAS.

[Read before the San Antonio Medico-Social Club.]

GENTLEMEN of the San Antonio Medico-Social Club:—I wish to state that it is a task for me to attempt to write a paper, or to read one, after it has been written, and it is only duty that ever impels me to such an undertaking.

Our genial host delights in relating experiences, and several of our fellows, also, have the happy faculty of writing with easy grace. There is one consolation, however, if I am the first to be honored, I can, in the future, enjoy our meetings more fully as the nervous apprehension of being called on for a paper will not mar my pleasure. It is either a defect in my nature, or, as one of my friends said when I refused to join a few of them in the great national game, poker, telling him I had never seen the game played; he replied that my education had been neglected. The feeling, therefore, of a sense of duty prompted me to comply with the request of our chairman, and it will be the subject of my disjointed paper, namely: *Our Duty as Physicians.*

For convenience, I will confine myself to three divisions: our duty to our patients; our duty to each other, and our duty to ourselves.

In a general way, our duty to our patients comes first, and, with a few exceptions, nothing should interfere, or deter us from its faithful performance. Ours is, indeed, a noble and high calling, and demands the entire time and talent of him who chooses medicine for his profession. When we see the physical suffering and deformity, followed by moral depravity and, oftentimes, crime, the question for each of us to answer is: What can be done to prevent or lessen this state? Our lawmakers have multiplied statutes until it would require a train to carry them all. Our philanthropists have built hospitals, infirmaries, homes and asylums; the preachers have worn themselves out preaching against vice, and all to no purpose. We know that never in the history of the world, were the penitentiaries, jails, asylums, hospitals, and "homes," so crowded. How to improve the race physically is a question that concerns us directly, and one that each of us is responsible for to the extent of his ability, or influence. In the slow process of evolution from a lower to a higher plane, there have never been any radical changes; but just like tissue repair by cell action, each cell adding its mite. Therefore each of us may influence those we are called to treat, showing them how to live, and how their lives may affect those unborn, for two, and sometimes, four generations. Since we have learned that disease itself is not inherited nor transmitted, it seems to me a wider field of usefulness has been opened to us, and a correspondingly greater responsibility.

If we take a few of the diseases that are so common, and study their far-reaching effects, it is simply appalling. Let us look for a few moments at those over which our influence should have some beneficial effects, and yet, seemingly, they are becoming more prevalent. I will put first in importance dyspepsia, including by this term all the different forms. This implies malnutrition of all the tissues of the body; and the earlier in life, of course, the more far reaching and disastrous the results. This condition, or rather this predisposition, is inherited, and affects, to a marked degree, the life and happiness of every child born of dyspeptic parents, and probably the tendency is increased tenfold in the next generation. Since we know that each individual is provided with resisting force, by cell action, to repel or overcome most diseases, provided assimilation and elimination are in perfect condition, we realize how important for the welfare of a nation it is that the people should know how to live. If the great body of physicians of the world, in their individual capacity, would use their influence in teaching their patients how to eat, what to eat, how to dress, and how to exercise, we would be laying the foundation stone, so to speak, for a grander race of people than has ever inhabited our globe.

It seems to me that the physician has been overzealous in studying the supposed curative effects of drugs, and neglected how to increase the resisting power of the patients who confidingly send for him to minister to their physical ills. It has been demonstrated conclusively that cirrhosis of the liver and Bright's disease of the kidneys, in many instances, are the result of fermentative processes in the alimentary tract, and effete material in the circulation. Haig has shown us how uric acid and urea accumulate in the system, and how certain foods tend to increase this; also the number of diseases that are the result, or are markedly influenced by this condition. Here we see how important it is to teach proper living, thereby preventing many of the common everyday ills that are incurable by medicines, and tend to mar the pleasure and usefulness of so large a per cent. of the people.

We can readily see how a person of low resisting power will contract diseases whilst his brother or neighbor, exposed to the same causes, escapes unharmed.

Tuberculosis comes next in my opinion. In regard to this disease we have certainly been derelict in our duty to those afflicted, their associates, and to posterity. We should teach them the dangers of the sputa, how to destroy or dispose of it, and show them what a crime it is to marry, or have children if already married. Syphilis, too, claims our earnest attention; for, it has spread over the globe, and claims its victims by the millions. It is estimated that in the city of Berlin, one out of every nine of its inhabitants is syphilitic. We have been alert in learning how to cure, or I should say, treat this dread disease; but, what have we done to stamp it out, or prevent it?

I will not weary you with enumerating the long list, but it seems to me, for the cause of humanity, we should use our influence, individually and collectively, to prevent the spread of these two diseases. Why not pass laws prohibiting the marriage of parties afflicted with such diseases as tuberculosis, syphilis or gonorrhœa? Insanity and epilepsy should also be included. Have a law passed as suggested by a recent article in the *American Journal of Obstetrics and Diseases of Children* compelling a man to produce a certificate of health before being granted a licence to marry.*

---

*The TEXAS MEDICAL JOURNAL has advocated the same course; it is one of its hobbies.—ED.

It seems to me such a law would be both wise and humane. Look at our army of invalid women; it is the exception to-day to see a sound, healthy woman who is the mother of three or more children. Is it not a reflection upon our boasted civilization, and upon our proud skill? Have we done our duty in speaking in no uncertain tone about the pernicious, far-reaching evils of the corset and skirts dragging from the waist? We have a host of men vieing with each other in publishing results of how many hyterectomies and ovariotomies they have performed within the year; but would it not be better to teach the girls how to avoid prolapse of stomach, bowels, womb and ovaries, by sensible dress, healthful exercise, and a well rounded out physical development? I would not have those men less skillful in their efforts to save life and restore the wife and mother; but this is an age of prevention; and whilst the family physician who honestly and intelligently teaches the families he is called to treat, how to live correctly, may not win fame or great riches, yet who can deny that his life has been the most successful of the two? The far reaching effects of his influence are beyond calculation.

Our duty to each other. It would seem useless to mention or make any remarks about a subject that could be expressed in that wise command or proverb: Do unto others, etc. Yet, when we reflect upon the social relations among physicians, we are impressed with the fact that something is radically wrong. It seems to me an opportune time at this, our first meeting, to consider this question. We do not command the respect of the people which is justly ours, nor exert but a fraction of the influence we should command. Why should we fight each other, and let the tale-bearing patient influence or bias our feelings and cause estrangements? If we would always rebuke the patient who flatters us and abuses his former physician, and probably has not paid him for long and faithful service, it would teach him a useful lesson, and increase his respect. Who can estimate the effect for our good, if those of us constituting this society should be loyal each to the other on all occasions? There is some good in every man, and many faults in the best men; therefore, let us be charitable in our criticisms. If I were asked to give the cause of the present condition in two words, would say "jealousy and bigotry."

Then, again, there are always black sheep in every community; men without honor, who will resort to all the low tricks possible, and slander their competitors in order to gain practice. How should we deal with this class has always been a vexed question. It is useless to abuse them, for there are times when duty demands us to see cases with them. Let us, then, as fellows of this society, resolve to be united in upholding the honor of each other and of the cause we represent. We can influence the entire profession of the city, and be a medium for good which will reach out in many ways, and benefit each one of us, both directly and indirectly. We are told by the wisest of men that our chiefest duty is to get wisdom, and with all our getting, to get understanding. In the struggle for practice, and with the great majority, for decent support, we neglect our duty to self. Our lives are, in many respects, unenviable, and often devoid of social pleasures or healthful recreation.

We send our patients to the mountains or seashore, oftimes leaving our bills unpaid, to enable them to take the trip; and thereby necessitating our staying at home all summer for lack of a few dollars to get a much needed change. There is so much at stake when we consider the probability of failing health, that it behooves us to look well after our own physical welfare, that each may be able, at all times, to do his best. It is given to few the inspiration or genius to reach the heights of Sims or Pasteur; but our lives are not less important or honorable. It is said that to estimate a man's wealth, you must first know the state of his conscience and health. Our duty is to save.

